# Supplementary material for: Slow light topological photonics with counter-propagating waves and its active control on a chip
Source: Nat Commun. 2024 Jan 31;15:926. doi: 10.1038/s41467-024-45175-5 (PMC10830473; doi:10.1038/s41467-024-45175-5)
Supplement: Supplementary file 1 — Supplementary Information [file 41467_2024_45175_MOESM1_ESM.pdf]

Slow Light Topological Photonics with Counter-Propagating Waves and its Active Control on a Chip

Abhishek Kumar[1,2 †], Yi Ji Tan[1,2 †], Nikhil Navaratna[1,2], Manoj Gupta[1,2], Prakash Pitchappa[3], Ranjan Singh[1,2 *]

[1]*Division of Physics and Applied Physics, School of Physical and Mathematical Sciences, Nanyang Technological University, Singapore 637371*
[2]*Centre for Disruptive Photonic Technologies, The Photonics Institute, Nanyang Technological University, Singapore 639798*
[3]*Institute of Microelectronics, Agency for Science, Technology and Research, 2 Fusionopolis Way, Singapore 138634*

** Corresponding Author- ranjans@ntu.edu.sg*

† These authors contributed equally

Supplementary Information

S1: Eigenvalue analysis of Valley Photonic Crystals (VPC)

a. Photonic band structure

Valley photonic crystal (VPC) consists of equilateral triangular holes arranged in hexagonal lattice with periodicity $a = 260\ \mu\text{m}$. The unit cell of VPC constitute two triangular holes facing each other, shown in Supplementary Figure 1a. When the side length of the triangular holes is equal, there exists a pair of degenerated Dirac points at K and K' valleys in the photonic band diagram as shown in Supplementary Figure 1a. These Dirac points are protected by inversion and time reversal symmetry. Therefore, breaking either inversion or time reversal symmetry can lift the degeneracy of the Dirac points. In our case, we break the inversion symmetry by setting the side length of triangular holes to be unequal (i.e., $l_1 \neq l_2$), opening the photonic bandgap as shown in Supplementary Figure 1b. Note that the photonic band-structure is identical for both type of unit cells: Type A and Type B, characterized by the sign of δ, defined as $\delta = l_1 - l_2$. However, inspecting the Floquet eigenstate near the K and K' valleys reveal a self-rotating vortex feature in the phase of Floquet eigenstate. Moreover, for a given unit cell, the chirality of phase vortex is opposite at K and K' valley. The difference in Type A and Type B unit cells are more obvious when we analyze the distribution of phase vortex. At a given valley (either K or K'), the chirality of phase vortices is also opposite for these unit cells.

We consider VPC that exhibits photonic bandgap for transverse electric (TE) mode, where the electric fields are confined in-plane (xy-plane). The out-of-plane magnetic field (H_z) component acts as the Floquet eigenstates of VPC. Calculating the Berry curvature distribution for 2D Type A and Type B unit cell using $\Omega(\mathbf{k}) = \frac{\partial A_y}{\partial k_x} - \frac{\partial A_x}{\partial k_y}$ reveals complimentary behaviour at K/K' valley due to the chirality of magnetic phase vortex. Here, $\mathbf{A} = -i < u|\partial_{\mathbf{k}}|u >$ is the Berry connection with u being the periodic Floquet eigenstate of the lower band. For a given unit cell (either Type A or Type B), the Berry curvature is localized at K and K' valleys with opposite signs, as shown in Supplementary Figure 1c and 1d. The presence of time reversal symmetry

ensures the symmetrical distribution of Berry curvature, resulting in a zero Chern number, defined as $C_n = \frac{1}{2\pi}\oiint \Omega\, \mathrm{d}^2\mathbf{k}$, when integrated over the entire Brillouin zone. However, to establish the bulk-boundary correspondence, one can define a bulk quantity called valley-Chern number, which is similar to Chern number except the closed integration of Berry curvature is done only near to the K or K' valley.

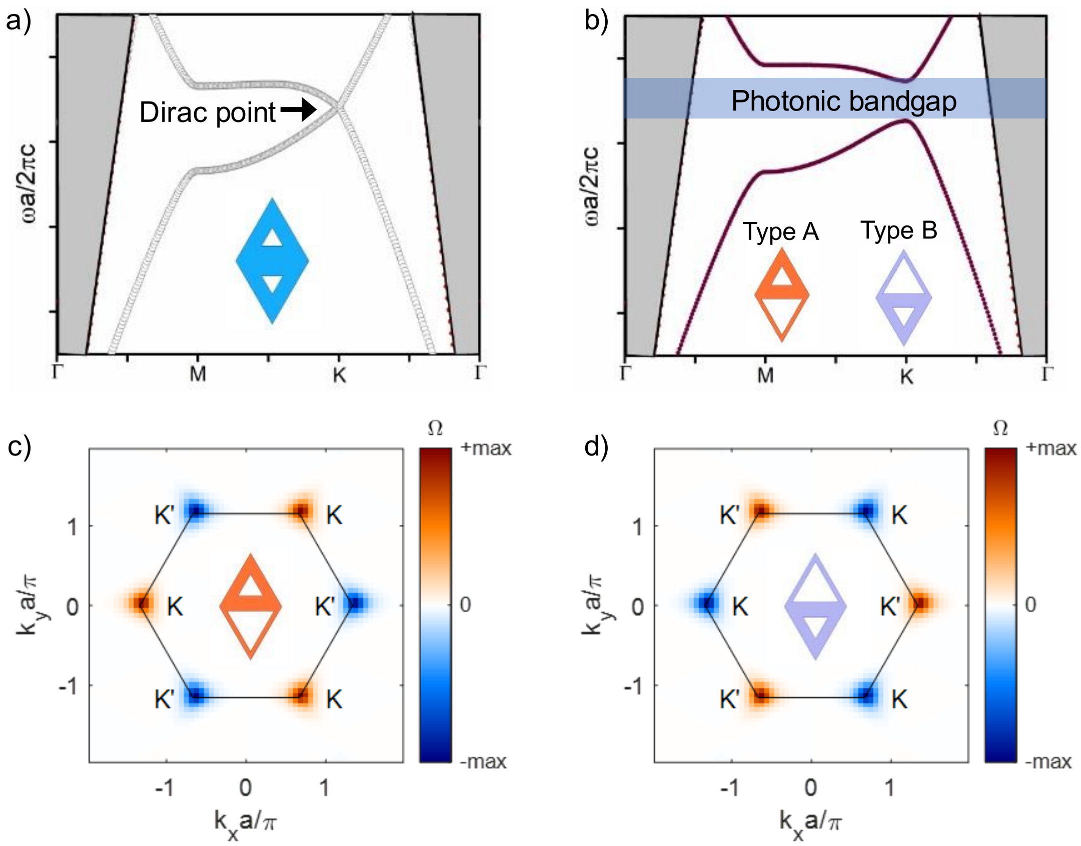

Supplementary Figure 1. Photonic TE band-structure of VPC. (a, b) Band structure of VPC for symmetric ($l_1 = l_2$) and asymmetric ($l_1 \neq l_2$) unit cell, respectively. The symmetric unit cell exhibits a Dirac point at K/K' valley, highlighted in (a). The photonic band diagram is the same for Type A and Type B unit cell, shown in (b). The grey region highlights the eigenmodes above the light line. **(c, d)** Berry curvature distribution of Type A and Type B unit cell, exhibiting localization at K and K' valley.

b. Identification of topological and trivial mode in bearded interface VPC waveguide

Regarding the assignment of trivial and topological bands, we have plotted the transmission through Z-shaped VPC waveguide. Supplementary Figure 2a and 2c show the projected band diagram for both Type I and Type II bearded interface with triangular air holes ($\nu = 3$). The solid and dashed lines in the diagrams highlight the topological and trivial modes, respectively. The topological and trivial modes are distinguished by examining the transmission spectra through Z-shaped Type I and Type II VPC waveguides, as shown in Supplementary Figure 2b and 2d. The high transmission observed within the frequency range (indicated by orange

shaded region) corresponding to the topological edge states signify the topological nature of the lower band for Type I bearded interface.

Similarly, for Type I bearded interface with hexagon ($\nu = 6$) and nonagon ($\nu = 9$) air holes, we observe that the lower bands exhibit topological characteristics, as evident from the high transmission within the frequency range covered by the topological edge states, as shown in Supplementary Figure 2e, 2f, 2i and 2j.

In case of Type II bearded interface with hexagon and nonagon air holes, where the radii (r) of air holes are $r > \frac{a}{2\sqrt{3}}$, they lead to a physically separated photonic crystal with an air-slot waveguide at the bearded interface. However, the presence of overlapping air holes (in the case of hexagon and nonagon) forming an asymmetric air-slot waveguide allows the backwards coupling of wave at the bends due to the propagation of the edge states along the domain wall. To address this issue, a composite bearded-zigzag interface junction was proposed in Opt. Express 30, 33035-33047 (2022).

Supplementary Figure 2g and 2k show the projected band diagram for the Type II bearded interface and zigzag interface. The topological edge modes are highlighted by the blue solid line, while the edge modes corresponding to zigzag interface are depicted by the black solid line. In projected band diagram, the region covered exclusively by the topological edge modes is highlighted by the blue shaded region.

Supplementary Figure 2h and 2l depict the transmission spectra through the composite interface with 60° bends constructed using hexagon and nonagon air holes, respectively. The high transmission observed within the frequency region highlighted by the blue shaded region confirms the topological nature of the lower and upper bands.

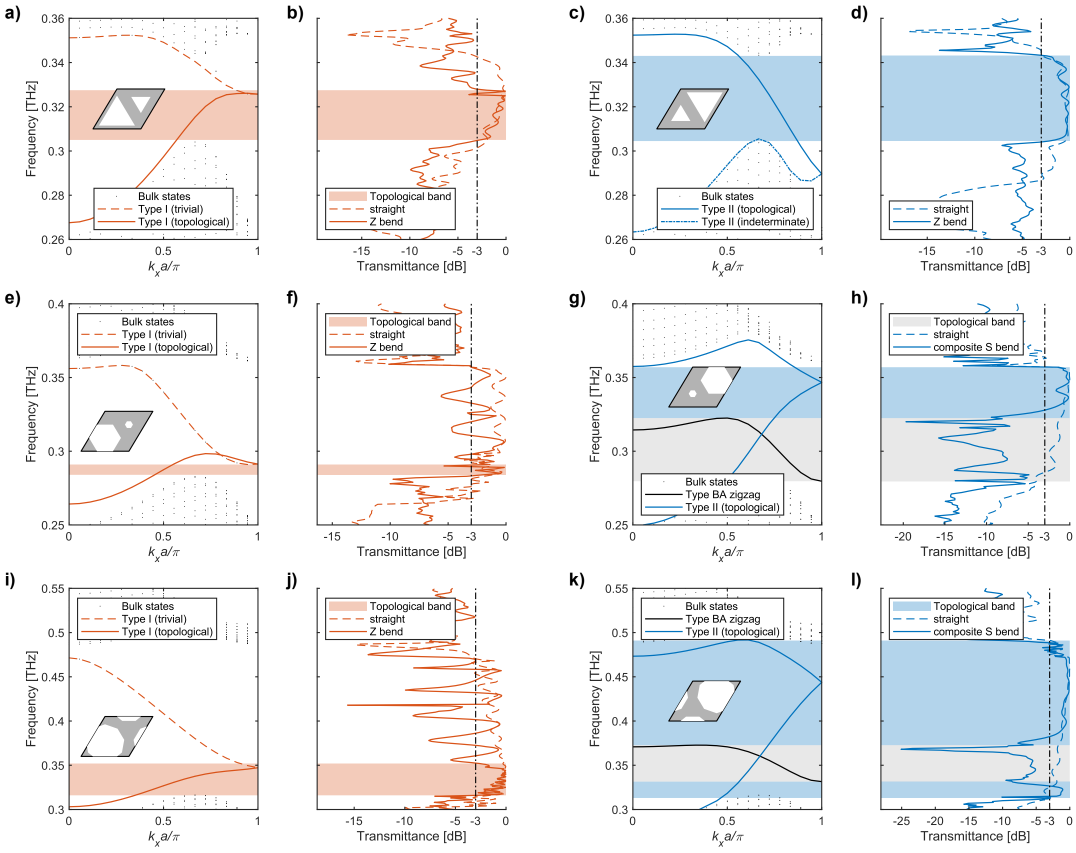

Supplementary Figure 2. Identification of topological and trivial modes in VPC waveguide. (a, b) Projected band diagram and transmission spectrum for Type I bearded interface with triangular air holes ($\nu = 3$), respectively. **(c, d)** Projected band diagram and transmission spectrum for Type II bearded interface with triangular air holes ($\nu = 3$), respectively. **(e, f)** Projected band diagram and transmission spectrum for Type I bearded interface with hexagon air holes ($\nu = 6$), respectively. **(g, h)** Projected band diagram and transmission spectrum for Type II bearded interface with hexagon air holes ($\nu = 6$), respectively. **(i, j)** Projected band diagram and transmission spectrum for Type I bearded interface with nonagon air holes ($\nu = 9$), respectively. **(k, l)** Projected band diagram and transmission spectrum for Type I bearded interface with nonagon air holes ($\nu = 9$), respectively.

S2: Valley kink states at zigzag interface

Building the VPC waveguide by stacking Type A and Type B unit cell to construct zigzag interface supports only forward or backward Poynting vector at a given K/K' valley. This occurs due to the locus of magnetic phase vortex that gives rise to only one-way propagating valley kink states. Supplementary Figure 3 shows the simulated Poynting vectors overlaid on zigzag interface VPC waveguide. We can observe that there exists only forward propagating Poynting vector away from the interface.

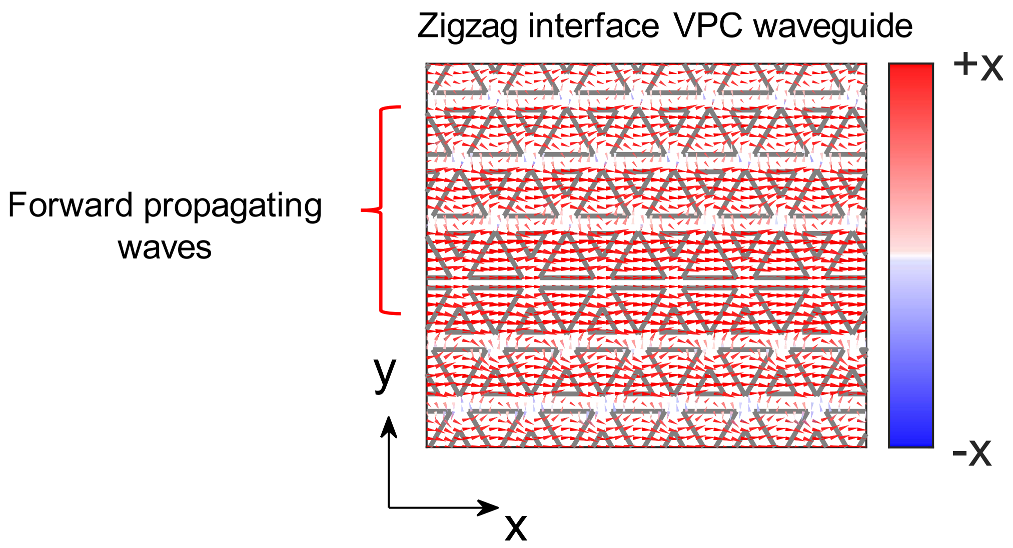

Supplementary Figure 3. Forward propagating kink state of zigzag interface VPC waveguide at K valley. The arrows show the Poynting vectors, where red arrows illustrate the power flow (S_x) in the $+x$ direction.

S3: Valley kink states at bearded interface

Constructing the VPC waveguide with bearded interface allows the existence of counter-propagating waves away from the domain wall as shown in Supplementary Figure 4. The interaction of these counter-propagating waves with forward propagating waves give rise to slow light feature in VPC waveguide. In Supplementary Figure 4, the red and blue arrows highlight the Poynting vector flow of forward and counter-propagating waves, respectively.

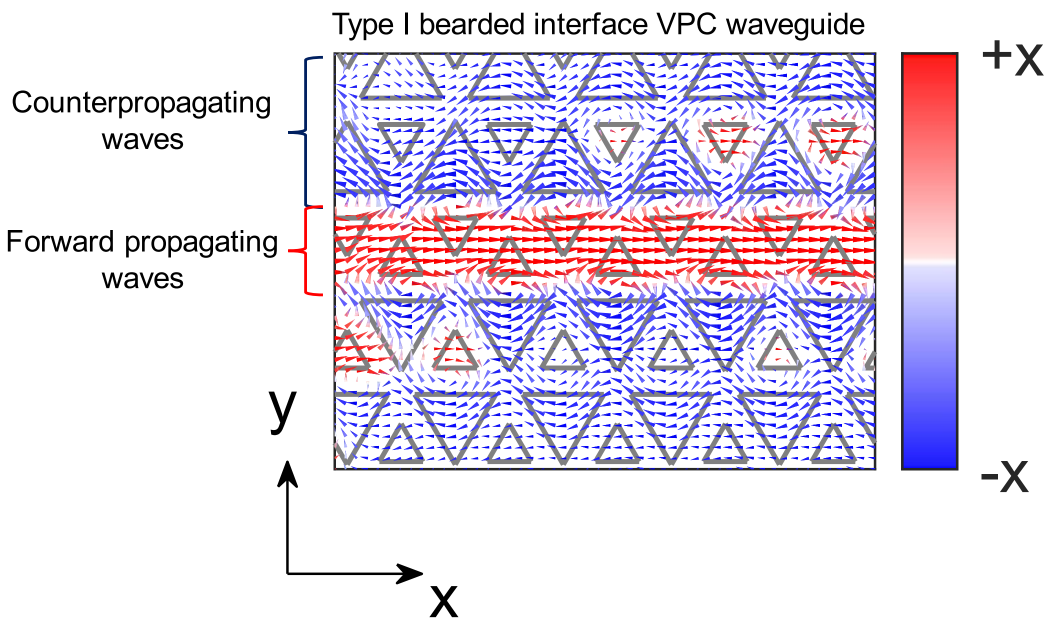

Supplementary Figure 4. Counter-propagating kink state of Type I bearded interface VPC waveguide at band edge. The arrows show the Poynting vectors, where red arrows illustrate the power flow (S_x) in $+x$ direction, while blue arrows represent counter-propagating power flow in $-x$ direction.

S4: Tuning slowness in VPC waveguide by interface engineering

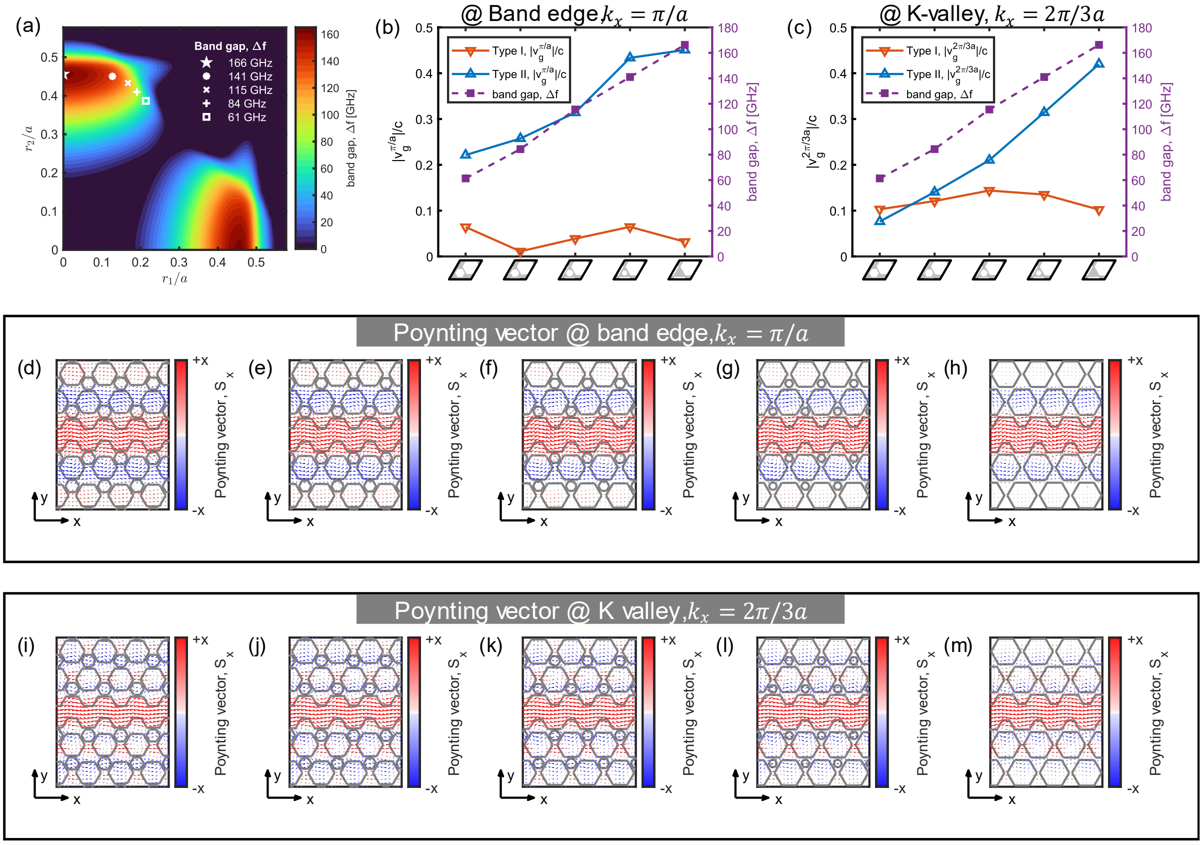

Supplementary Figure 5. Tuning the slowness of VPC via bandgap engineering. (a) The bandgap of VPCs as a function of air hole radii (r_1, r_2) for hexagonal air holes $(\nu = 6)$ of period $a = 260\ \mu\text{m}$. The colormap indicates the strength of bandgap, while markers represent the selected unit cells with increasing bandgap. The specific radii (r_1, r_2) of the air holes in these chosen unit cells are as follows: $\left(\frac{0.37}{\sqrt{3}}a, \frac{0.67}{\sqrt{3}}a\right)$, $\left(\frac{0.33}{\sqrt{3}}a, \frac{0.71}{\sqrt{3}}a\right)$, $\left(\frac{0.29}{\sqrt{3}}a, \frac{0.75}{\sqrt{3}}a\right)$, $\left(\frac{0.22}{\sqrt{3}}a, \frac{0.78}{\sqrt{3}}a\right)$ and $\left(0, \frac{0.79}{\sqrt{3}}a\right)$, with corresponding bandgap (Δf) values 61 GHz, 84 GHz, 115 GHz, 141 GHz and 166 GHz, respectively. **(b)** Band edge group velocity $(v_g^{\pi/a})$ is calculated for Type I (red solid line) and Type II (blue solid line) bearded interface VPC waveguides, shown on the left Y-axis. The bandgap corresponding to the selected unit cells is shown on the right Y-axis (purple line). **(c)** Group velocity extracted at projected K value (i.e., $k_x = 2\pi/3a$) for Type I and Type II bearded interface VPC waveguides, shown on the left Y-axis. **(d-h)** The locus of the Poynting vector extracted at $k_x = \pi/a$. The bandgap increases from 61 GHz to 166 GHz from left to right. **(i-m)** The locus of the Poynting vector is extracted at K valley i.e., $k_x = 2\pi/3a$. The bandgap increases from 61 GHz to 166 GHz from left to right.

Altering the bandgap allows us to tune the transverse decay length of the topological kink modes perpendicular to the interface, which affects the coupling strength between forward and counter-propagating waves. This concept is illustrated in Fig. 2 of the manuscript, where we achieve the bandgap tunability by changing the shape of air holes from triangle to nonagon. To further emphasize this, we performed an extensive investigation of the bandgap and group velocity (v_g). For this purpose, we fixed the air hole shape to be hexagon $(\nu = 6)$ and varied the air hole radii (r_1, r_2) for hexagonal air holes $(\nu = 6)$ of period $a = 260\ \mu\text{m}$. Supplementary Figure 5a presents the bandgap of VPCs, with colormap representing the strength of the bandgap. We selected the unit cells with increasing bandgap, as marked in Supplementary

Figure 5a. The specific radii (r_1, r_2) of the air holes in these chosen unit cells are as follows: $\left(\frac{0.37}{\sqrt{3}}a, \frac{0.67}{\sqrt{3}}a\right)$, $\left(\frac{0.33}{\sqrt{3}}a, \frac{0.71}{\sqrt{3}}a\right)$, $\left(\frac{0.29}{\sqrt{3}}a, \frac{0.75}{\sqrt{3}}a\right)$, $\left(\frac{0.22}{\sqrt{3}}a, \frac{0.78}{\sqrt{3}}a\right)$ and $\left(0, \frac{0.79}{\sqrt{3}}a\right)$, with corresponding bandgap (Δf) 61 GHz, 84 GHz, 115 GHz, 141 GHz and 166 GHz, respectively.

There is gradual decrease in the strength of the counter-propagating waves with an increase in bandgap (from left to right), which results in an increase in $v_g^{\pi/a}$ for Type II bearded interface. Moreover, to demonstrate the universality of the underlying mechanism of slow light in bearded interface VPC waveguide, which arises from the interaction between forward and counter-propagating waves, we calculated the group velocity at the projected K valley (i.e., $v_g^{2\pi/3a}$ where $k_x = 2\pi/3a$), as shown in Supplementary Figure 5c. Similar to the previous case, there is a lack of correlation between $v_g^{2\pi/3a}$ and the bandgap for Type I bearded interface while $v_g^{2\pi/3a}$ increases with the bandgap for Type II interface. This observation is further supported by the Poynting vector plot shown in Supplementary Figure 5i to 5m, where increasing the bandgap reduces the strength of counter-propagating waves, leading to an increase in group velocity ($v_g^{2\pi/3a}$).

S5: Experimental setup

Complex S-parameters are measured using Vector Network Analyzer (VNA) to obtain the transmission (S_{21}) and group delay. The VNA measurement setup features a Keysight N5222B network analyzer which generates signals in the frequency range 10 MHz to 26.5 GHz. These frequencies are then upconverted to the WR-2.2 frequency range using WM-570 VNAX frequency extension modules. The WR-2.2 VNA setup can measure response starting from 320 GHz. The system is then calibrated using a SOLT (Short-Open-Load-Through) waveguide calibration procedures adhering to WR-2.2 standards. Calibration accounts for system non-idealities and enables accurate measurements of VPC chip response.

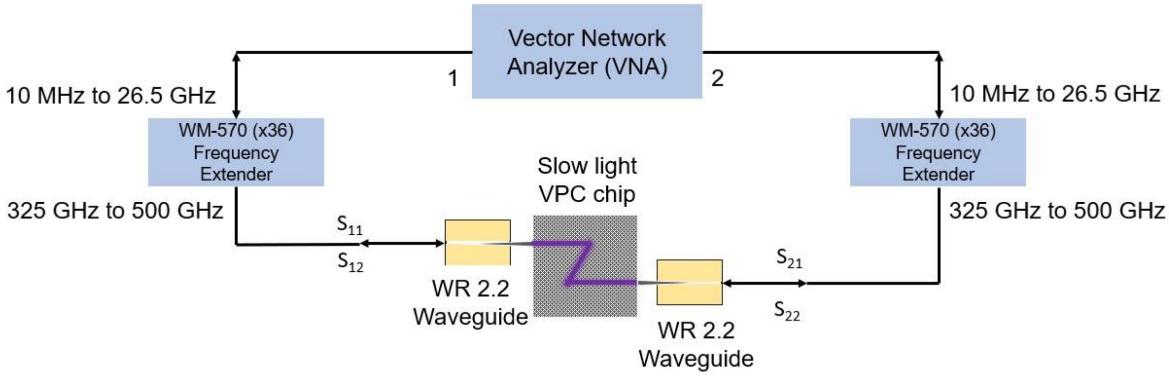

Supplementary Figure 6. Schematic of experimental setup. The VPC chip is inserted between WR 2.2 waveguides, where the Vector Network Analyzer (VNA) measures the response from 320 GHz to 500 GHz with the help of WM-570 frequency extension modules.

S6: Optical image of VPC chip

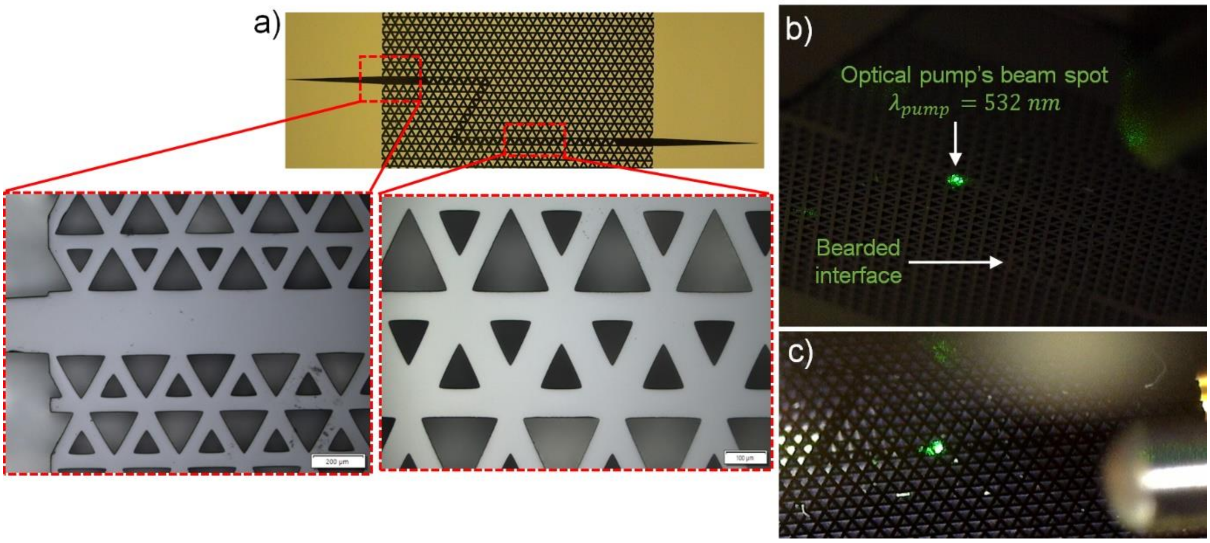

Supplementary Figure 7. Optical images of the fabricated VPC chip. (a) Optical image of the Type I bearded interface VPC waveguide chip with Z bend, along with the microscope image of the taper coupler region (bottom left) and the bearded interface region (center bottom). **(b, c)** Optical image of the bearded interface VPC waveguide chip under optical illumination using a continuous wave laser of wavelength 532 nm, where the optical beam spot is focused onto the bearded interface.

Supplementary Figure 8 depicts the optical image of the fabricated VPC waveguide chip, where the bearded interface Type I is highlighted by a blue shaded region. The right image shows the simulated Poynting vector overlaid onto the optical image for the highlighted rectangular region in the left image.

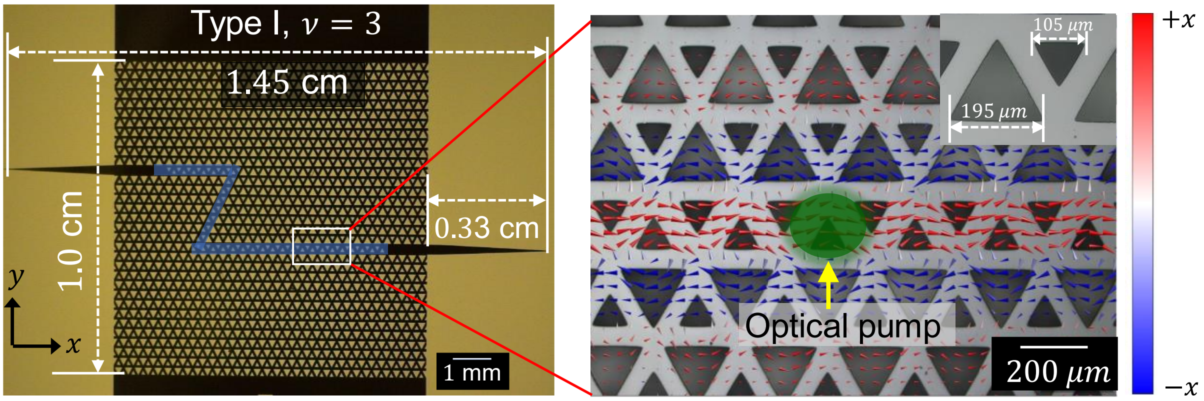

Supplementary Figure 8. Optical image of the fabricated Type I bearded interface VPC chip. The domain wall of the VPC waveguide is highlighted by blue shaded regions. Adiabatic input and output couplers are attached to couple the terahertz waves in and out of the VPC chip. In the right image, simulated Poynting vector (S_x) is overlaid onto the optical image of the rectangular region marked in the left image. Translucent green dot represents the focused optical pump region, while the inset highlights the unit cell of VPC marked with the dimensions of triangular holes.

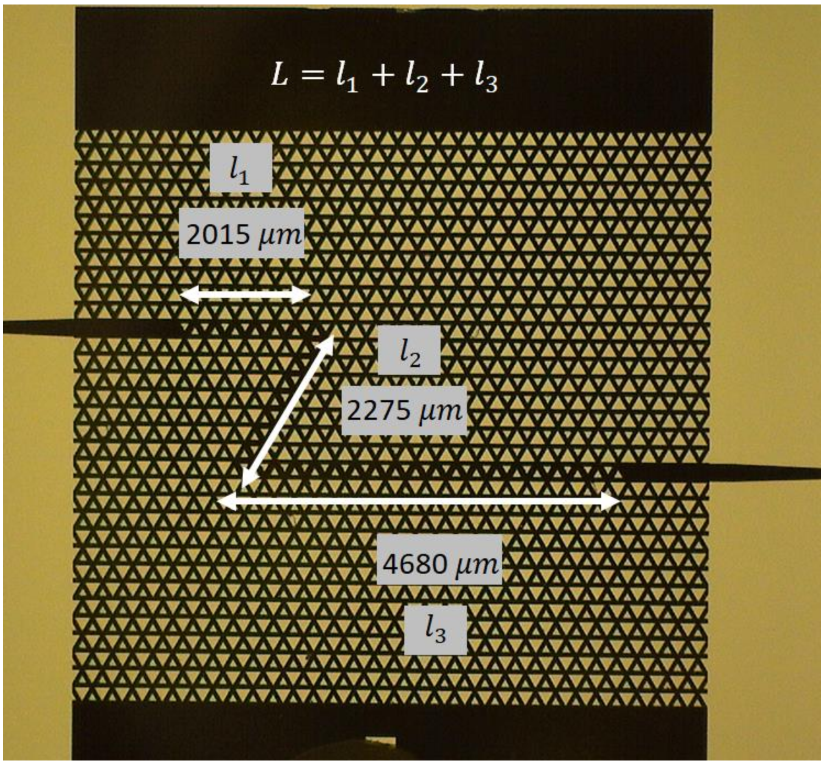

Supplementary Figure 9. The fabricated Type I bearded interface VPC waveguide. The length of the bearded interface is defined between the taper coupler as $L = l_1 + l_2 + l_3 = 8.97$ mm as shown.

S7: 3D simulation in CST Microwave Studio

To emulate the experimental conditions, we performed a full 3D simulation in CST Microwave Studio. The simulation setup, depicted in Supplementary Figure 10a, includes adiabatic couplers designed at both the input and output facets of the VPC waveguide. To couple the terahertz wave into the VPC waveguide, we used a rectangular port (port 1 in Supplementary Figure 10a) connected to the WR 2.8 metallic waveguide. The output signal was collected from the output port (port 2 in Supplementary Figure 10a) using an identical WR 2.8 metallic waveguide, as depicted in Supplementary Figure 10a.

To emulate the effects of optical pumping, we defined a circular region (highlighted in red in Supplementary Figure 10a) within the VPC waveguide, spanning a few unit cells of bearded interface with variable silicon conductivity. To ensure high accuracy in simulation, we implemented sufficiently dense meshing, particularly in the region where silicon conductivity varies, as shown in Supplementary Figure 10b and 10c.

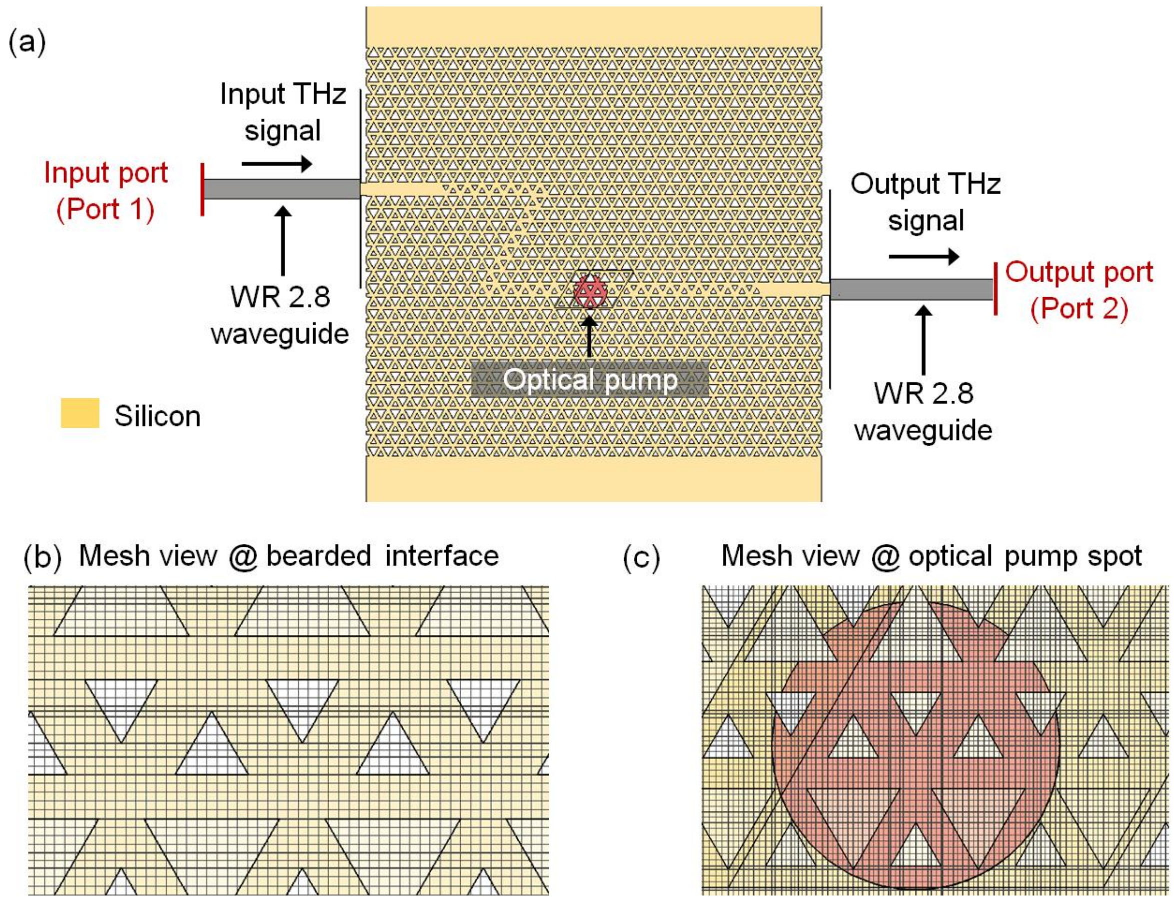

Supplementary Figure 10. 3D simulation setup in CST Microwave Studio. (a) Schematic of Type I bearded VPC waveguide with $\nu = 3$ in CST simulation environment. Adiabatic couplers are utilized to efficiently couple the THz waves in and out of the VPC waveguide. Rectangular ports (port 1 and port 2) are connected to WR 2.8 waveguides to launch and receive the THz signals. The red region in the diagram represents silicon with variable conductivity **(b, c)** The mesh views show a close-up of the bearded interface and the spot where the optical pump (i.e., region with variable silicon conductivity) is located, respectively. The entire simulation is carried out using a time domain solver.

S8: Plane wave expansion (PWE) method

a. Mathematical formulation

Plane wave expansion (PWE) method reformulates the time harmonic Maxwell's equation into an eigenvalue problem. Due to this, it is possible to calculate the band structure of VPC unit cells and projected band diagrams of VPC waveguide. In Fig. 2a to 2c of the manuscript. the projected band diagram is calculated using the PWE method. The PWE method is rigorous and provide numerically exact solution of Maxwell's equation. The accuracy of PWE is only limited by the number of plane waves ($m_{\max}$, $n_{\max}$) used in the Fourier expansion of the dielectric function of photonic crystal's unit cell.

The time harmonic Maxwell's equation for a non-magnetic material can be expressed as

$$\frac{1}{\varepsilon_r(\mathbf{r})}\nabla \times \left(\nabla \times \tilde{\mathbf{E}}(\mathbf{r})\right) = \frac{\omega^2}{c^2}\tilde{\mathbf{E}}(\mathbf{r}) \qquad \text{S1}$$

$$\nabla \times \left[\frac{1}{\varepsilon_r(\mathbf{r})}\left(\nabla \times \tilde{\mathbf{H}}(\mathbf{r})\right)\right] = \frac{\omega^2}{c^2}\tilde{\mathbf{H}}(\mathbf{r}) \qquad \text{S2}$$

Here, $\tilde{\mathbf{E}}(\mathbf{r})$ and $\tilde{\mathbf{H}}(\mathbf{r})$ are the electric and magnetic fields, $\varepsilon_r(\mathbf{r})$ is the relative permittivity, ω is the angular frequency and c is the speed of light in free space. For the 2D VPC, TE mode has $E_z = H_x = H_y = 0$. Therefore, we can simply solve for the out-of-plane component of magnetic field (H_z) in eqn. S2. By applying simple algebraic calculations, eqn. S2 can be transformed into an eigenvalue problem, described by

$$-\left[\frac{\partial}{\partial x}\left(\frac{1}{\varepsilon_r(\mathbf{r}_\parallel)}\frac{\partial}{\partial x}\right)+\frac{\partial}{\partial y}\left(\frac{1}{\varepsilon_r(\mathbf{r}_\parallel)}\frac{\partial}{\partial y}\right)\right]H_z(\mathbf{r}_\parallel)=\frac{\omega^2}{c^2}H_z(\mathbf{r}_\parallel) \qquad \text{S3}$$

In eqn. S3, both the inverse dielectric function $\frac{1}{\varepsilon_r(\mathbf{r}_\parallel)}$ and H_z can be expressed as a sum of plane waves in the reciprocal lattice vector basis, given by:

$$\frac{1}{\varepsilon_r(\mathbf{r}_\parallel)}=\sum_{\mathbf{G}''_\parallel}\chi(\mathbf{G}''_\parallel)\mathrm{e}^{i\mathbf{G}''_\parallel\cdot\mathbf{r}_\parallel} \qquad \text{S4}$$

$$\mathbf{H}_{z,\mathbf{k}_\parallel}(\mathbf{r}_\parallel)=\sum_{\mathbf{G}''_\parallel}\mathbf{H}_{z,\mathbf{k}_\parallel n}(\mathbf{G}''_\parallel)\mathrm{e}^{i(\mathbf{k}_\parallel+\mathbf{G}''_\parallel)\cdot\mathbf{r}_\parallel} \qquad \text{S5}$$

Here, $\mathbf{k}_\parallel = k_x\hat{\boldsymbol{x}} + k_y\hat{\boldsymbol{y}}$ is in-plane wavevector and $\mathbf{G}''_\parallel = m\mathbf{b}_1 + n\mathbf{b}_2$ is reciprocal lattice vectors, with $\mathbf{b}_1$ and $\mathbf{b}_2$ as primitive reciprocal lattice vectors, where m and n are integers. $\chi(\mathbf{G}''_\parallel)$ is the Fourier coefficient of inverse dielectric function. Substituting eqn. S4 and S5 in eqn. S3, we get the following eigenvalue equation,

$$\sum_{\mathbf{G}'_\parallel}\left[(k_x+G_x)(k_x+G'_x)+(k_y+G_y)(k_y+G'_y)\right]\chi(\mathbf{G}_\parallel-\mathbf{G}'_\parallel)H_{z,\mathbf{k}_\parallel n}(\mathbf{G}'_\parallel)=\frac{\omega^2}{c^2}H_{z,\mathbf{k}_\parallel n}(\mathbf{G}_\parallel) \qquad \text{S6}$$

Solving eqn. S6 yields the band structure of VPC unit cell and projected band diagram of VPC waveguide. To ensure the convergence of results, we used 25 plane waves components for the computation of eigenfrequencies.

b. Simulation of group velocity (v_g) with photoexcitation emulation

The experimentally measured group delay (GD) is inversely proportional to the group velocity (v_g) of kink state. To emulate the behaviour of GD with optical pump power, we simulate v_g by introducing the non-zero imaginary part of refractive index of silicon in the PWE method. v_g is extracted from the projected band diagram. The non-zero value of imaginary component of refractive index qualitatively account for the loss induced by photoexcitation in experiment. To imitate the effect of small optical pump spot, the imaginary part of refractive index to silicon is only included to the vicinity of domain wall covering one row of unit cell as shown in Supplementary Figure 11. To ensure the convergence of the results, we used more than 100 plane wave components for the computation of eigenstate of projected band diagram.

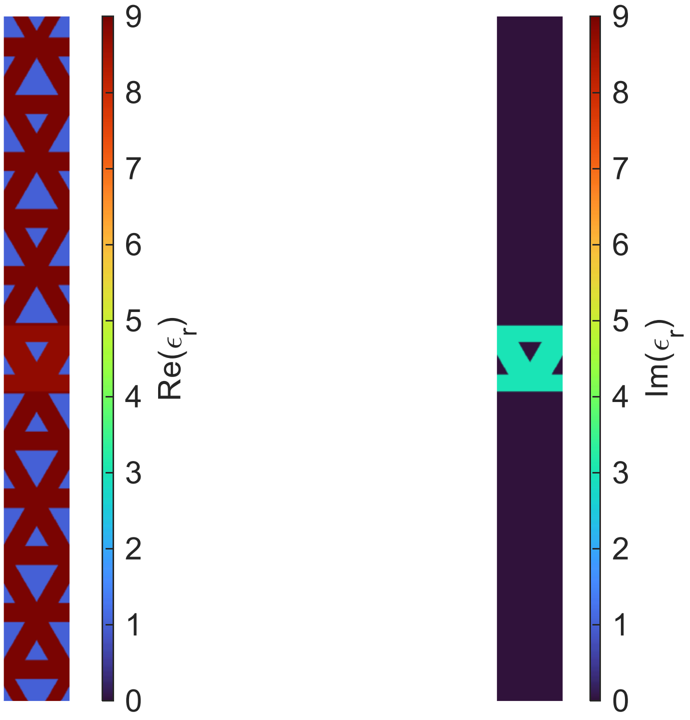

Supplementary Figure 11. Dielectric function of the VPC interface supercell with triangular air holes. Material loss, $n = 3.0, \kappa = 0.5, \varepsilon_r = (n + i\kappa)^2$, is introduced, covering one unit cell as shown.

Nonetheless, to highlight the unprecedented control on engineering the group index (n_g) in VPC chip, we performed a simulation by introducing the non-zero imaginary part (κ) of Si refractive index in the PWE simulation. The imaginary part of refractive index of Si is introduced near to the vicinity of domain wall covering one unit cell ($|y| < \pm\frac{a\sqrt{3}}{4}$). Supplementary Figure 12 depicts the simulated n_g at Brillouin zone edge as a function of κ. The right image of Supplementary Figure 12 shows the simulated v_g of the topological kink states calculated from the projected band diagram, where the colour gradient from red to blue highlights the variation of κ from 0 to 1.5, respectively, used in the PWE method. The square markers in Supplementary Figure 12 highlights the v_g at Brillouin zone edge (i.e., $v_g^{\pi/a}$). The n_g at Brillouin zone edge in Supplementary Figure 12 is then computed using $n_g = \frac{c}{v_g}$. The introduction of optical excitation in the form of nonzero imaginary refractive index of Si showcases not only the effective group velocity ($v_g^{\pi/a}$) tunability at the band edge at $k_x = \pi/a$, but also the tuning of v_g throughout the band from $k_x = 0$ to $k_x = \pi/a$ over a band of frequencies as shown in the right image of Supplementary Figure 12.

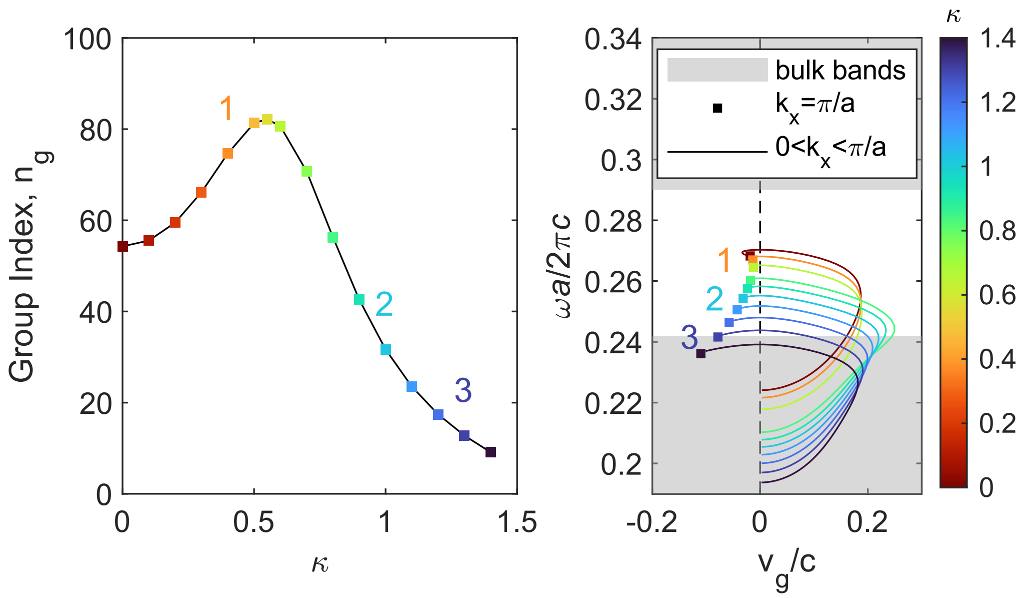

Supplementary Figure 12. Active tuning of group index (n_g), simulated by introducing non-zero imaginary part (κ) of Si refractive index in the PWE simulation for Type I bearded interface VPC. The unit cell is made of air holes with $\nu = 3$ ($l_1 = 0.65a, l_2 = 0.35a$; $a = 260\ \mu\text{m}$). The colour gradient from red to blue highlights the variation of κ from 0 to 1.5, respectively. The square markers highlight the v_g at Brillouin zone edge (i.e., $v_g^{\pi/a}$). The numerical labelling 1, 2 and 3 marks the one-to-one correspondence between n_g and $v_g^{\pi/a}$.

S9: Transport of kink state at various photoconductivity of silicon in VPC chip (2D COMSOL simulation)

To emulate the behaviour of optical pumping induced feature in the VPC slow light device, we performed frequency domain simulation in COMSOL. In our study, we conducted a 2D simulation using COMSOL Multiphysics to analyze the Poynting vector (power flow) in the VPC waveguide by locally varying the conductivity of silicon near to the bearded interface, as shown in Supplementary Figure 13a. Similar to 3D simulation (Supplementary Figure 10), the terahertz waves are coupled into the VPC waveguide using an input port connected to the WR 2.8 waveguide with an adiabatic taper coupler inserted, as depicted in Supplementary Figure 13a.

To replicate the behaviour of the WR 2.8 metallic waveguide in COMSOL, we employed perfect electric conductor (PEC) boundary condition at the edges (Supplementary Figure 13a). For the simulation process, we utilized the electromagnetic waves, frequency domain (ewfd) module of COMSOL Multiphysics. To ensure accuracy, we implemented user assigned mesh setting, and define the maximum and minimum mesh element sizes as $25\ \mu\text{m}$ and $5\ \mu\text{m}$, respectively. To emulate the 3D effect, we used effective index of silicon as 3.0 in the 2D COMSOL simulation.

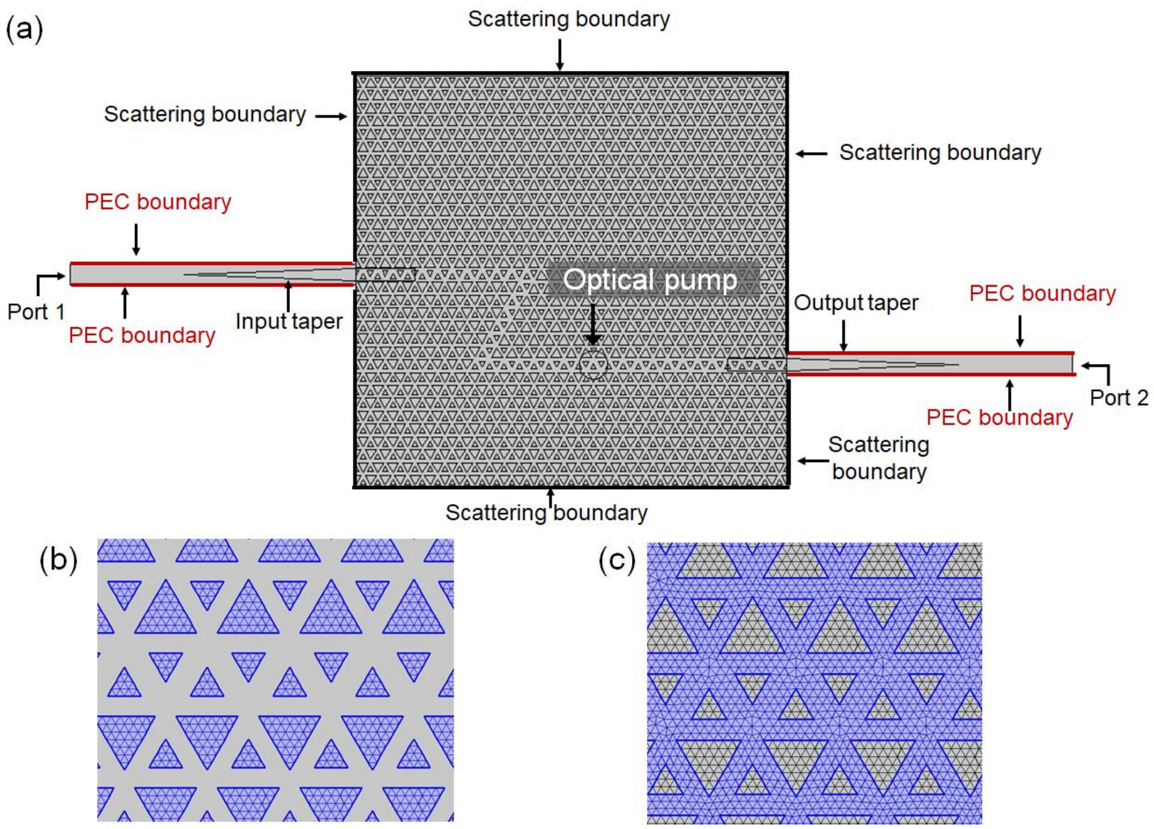

Supplementary Figure 13. 2D simulation setup in COMSOL Multiphysics. (a) Schematic of 2D simulation setup in COMSOL Multiphysics. Perfect electric conductor (PEC) is employed at the edges of rectangle to mimic the WR 2.8 metallic waveguide. Numeric ports are defined at both the input and output facets of the WR 2.8 waveguide. The VPC waveguide is surrounded by a scattering boundary condition to eliminate the effect of scattered waves. The black circle represents the area in VPC waveguide where silicon conductivity is varied. **(b, c)** The mesh views of air holes only and air holes with silicon area, respectively. To ensure accuracy, we implemented user assigned mesh setting, and define the maximum and minimum mesh element sizes as $25\ \mu\mathrm{m}$ and $5\ \mu\mathrm{m}$, respectively.

Supplementary Figure 14 shows the simulated transmission spectra from COMSOL Multiphysics. A bearded interface supports both topological and trivial mode due to the presence of glide symmetry. Building upon the ability of valley kink states to overcome sharp bends, we eliminate the contribution of trivial states in the transmission spectrum by constructing Z-shaped domain walls featuring two sharp bends. As a result, the transmission corresponding to the trivial mode is effectively suppressed in the waveguide with bends, leading to a reduced spectral bandwidth, as depicted by the red shaded region in Supplementary Figure 14.

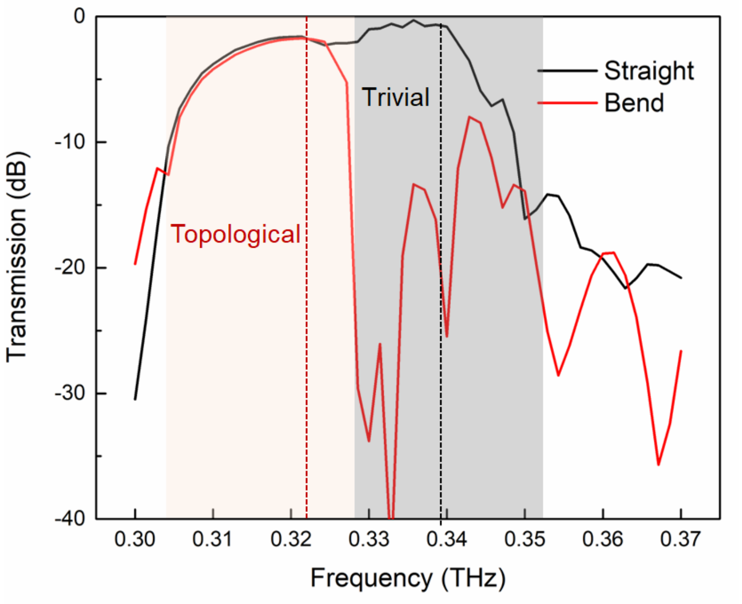

Supplementary Figure 14. Simulated transmission spectra from the VPC waveguide for both the straight (black solid line) and bend (red solid line) bearded interface configurations. In the bend waveguide**,** the transmission associated with the trivial modes is suppressed. The red and black shaded region represent the topological and trivial band, respectively.

Additionally, to explore the impact of photoexcitation on the transport properties of both topological and trivial modes, we conducted Poynting vector simulations using COMSOL. Within this analysis, we introduced a circular region spanning the bearded interface with varying silicon conductivity. The Poynting vectors were simulated at two distinct frequencies corresponding to topological and trivial modes, as highlighted by the vertical dashed line in Supplementary Figure 14.

Supplementary Figure 15 shows the simulated Poynting vector for different silicon conductivity values for both topological and trivial modes. The black circle encloses the region with variable silicon conductivity. Upon investigating the evolution of Poynting vectors for the topological mode (Supplementary Figure 15a to 15c), it becomes apparent that the kink state takes a detour around the circular region and retain its forward and counter-propagating waves after traversing the circular region. In contrast, for the trivial mode, there is a significant scattering and back reflection with an increase in silicon conductivity, as evident in Supplementary Figure 15d to 15f.

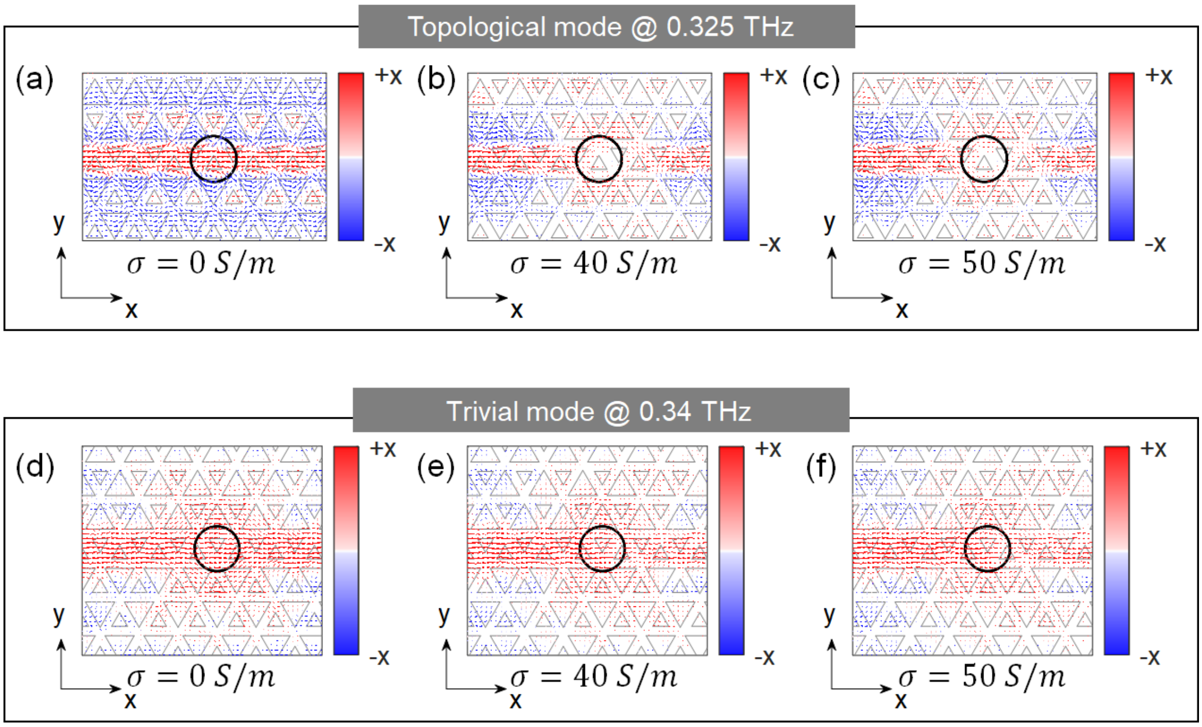

Supplementary Figure 15. Locus of Poynting vector simulated in COMSOL Multiphysics. (a-c) The Poynting vector is simulated for the frequency corresponding to the topological mode (i.e., 0.325 THz), marked by red dashed vertical line in Supplementary Figure 14. The region of the VPC with variable silicon conductivity is enclosed within a black circle. From left to right, the conductivity of silicon increases from 0 S/m to 50 S/m. **(d-f)** The Poynting vector is simulated for the frequency corresponding to the trivial mode (i.e., 0.34 THz), marked by the black dashed vertical line in Supplementary Figure 14. Similar to previous case, the region of VPC with variable silicon conductivity is enclosed within a black circle. The conductivity of silicon increases from 0 S/m to 50 S/m from left to right.

S10: Transmission and group delay (GD) measurements

To extract the transmission spectra, we calibrate the VNA setup initially. Post calibration, the VPC chip is gently inserted into the WR 2.2 waveguide with the help of DINO-Lite camera. Transmission (S_{21}) and group delay (GD) are directly obtained from the VNA. The measured phase data allows the calculation of group delay (GD).

$$\mathrm{GD} = -\frac{\mathrm{d}\phi}{\mathrm{d}\omega}$$

Here, ϕ is the phase data and ω is angular frequency. The group delay measurement trace portrays the time taken by each frequency to travel through the device being studied. It essentially converts the linear phase shift encountered by each frequency value to a constant that represents the average time delay attributable to propagation through the VPC chip. The group delay aperture, which defines the spacing between the frequency points where the phase is measured, is adjusted to yield optimum signal-to-noise ratio (SNR). For the results highlighted in this paper, the group delay aperture was 20 MHz. Supplementary Figure 16b shows the experimentally recorded transmission and GD spectra in the absence of optical pump, which shows good agreement with the 3D simulation (Supplementary Figure 16a). However, due to slight experimental misalignment and variations in the thickness ($\pm$ 25 μm)

of the fabricated VPC waveguide, there are minor differences observed in the transmission amplitude and GD (marked by an asterisk).

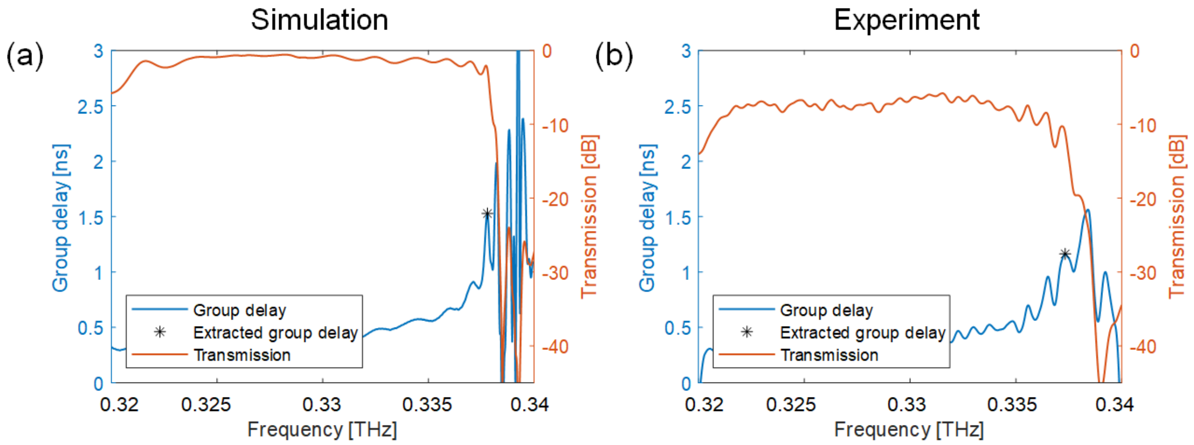

Supplementary Figure 16. Transmission and group delay (GD) spectra. (a, b) Simulated and experimental transmission (red solid line) and GD (blue solid line) spectra. The asterisk mark represents band edge frequency for which GD is extracted.

To ensure the consistency of our results, we conducted photoexcitation measurements multiple times by gradually increasing and decreasing the optical pump power. The results are depicted in Supplementary Figure 17, showing no variation in the measured GD values. This observation rules out the possibility of any hysteresis-type behaviour in our experiment.

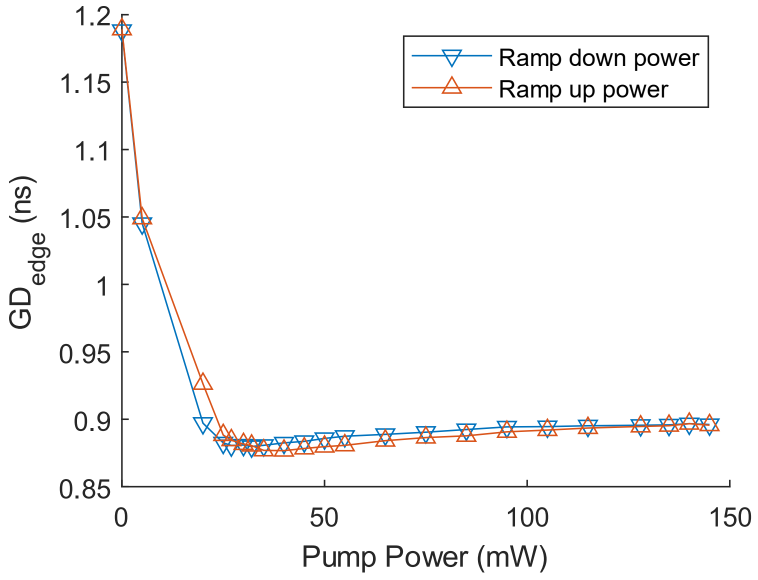

Supplementary Figure 17. Group delay (GD) as a function of optical pump power. The data points show the experimentally measured group delay at the transmission band edge as a function of optical pump power, as the pump power is gradually increased (ramp up power) and for gradually decreasing pump power (ramp down power).

S11: Transmission and group delay (GD) measurements

We conducted a thorough investigation into the influence of defects on slow light modes within our fabricated VPC waveguide (Type I, $\nu = 3$). To achieve this, we employed two distinct methods:

a. We varied the dimensions of triangular holes (Supplementary Figure 18a to 18c), leading to the introduction of fabrication defects.
b. Subsequently, we removed specific triangular holes from the waveguiding path (Supplementary Figure 18d to 18f).

To evaluate the consequence of these introduced defects, we computed the corresponding Poynting vectors using COMSOL Multiphysics. The results of these simulations are depicted in Supplementary Figure 18. In Supplementary Figure 18a to 18c, the dimensions of six triangular holes were varied, ranging from $l_1 = 0.4a/\sqrt{3}$ to $l_1 = 0.55a/\sqrt{3}$, where $a = 260\ \mu\text{m}$ represents the periodicity of unit cell. We selectively removed one to three triangular holes. Examining the locus of Poynting vectors in Supplementary Figure 18a to 18f reveal that the kink state takes a detour around the defect region while retaining its forward and counter-propagating moving waves characteristic even after traversing the region of geometrical imperfections, exhibiting robust transport behaviour.

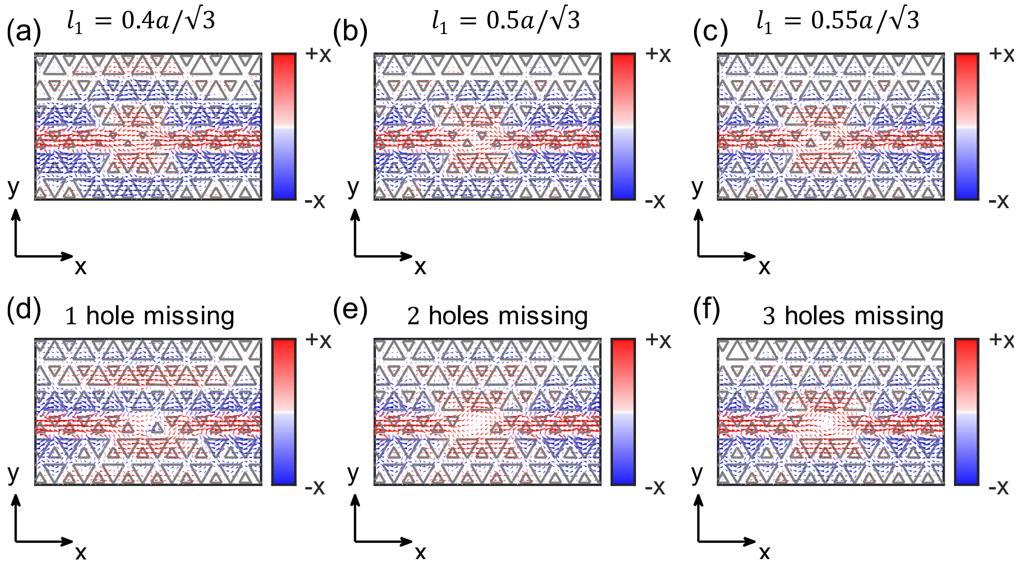

Supplementary Figure 18. Transport of slow light in VPC waveguide through defects. (a-c) The locus of Poynting vectors of slow light modes when the defects are introduced by varying the size of the triangular holes from $l_1 = 0.4a/\sqrt{3}$ to $l_1 = 0.55a/\sqrt{3}$, where $a = 260\ \mu\text{m}$ is the periodicity of unit cell. **(d-f)** The locus of Poynting vectors of slow light modes when the defects are introduced by removing the triangular holes.
