# Supplementary material for: Slow light topological photonics with counter-propagating waves and its active control on a chip
Source: Nat Commun. 2024 Jan 31;15:926. doi: 10.1038/s41467-024-45175-5 (PMC10830473; doi:10.1038/s41467-024-45175-5)
Supplement: Supplementary file 3 — Description of Additional Supplementary Files [file 41467_2024_45175_MOESM3_ESM.pdf]

Description of Additional Supplementary files

Supplementary Movie 1: Magnetic field profile of zigzag interface with triangular air holes, with the wavevector at projected K valley, showing only forward propagating waves.

Supplementary Movie 2: Magnetic field profile of Type I bearded interface with triangular air holes, with the wavevector at band edge, showing both forward and counter propagating waves.

Supplementary Movie 3: Magnetic field profile of Type I bearded interface with Z bend for triangular air holes (left), and the corresponding transmission spectrum (right), showing the existence of counter-propagating waves over a finite frequency bandwidth with wavevectors from the projected K valley to the band edge.
