# Supplementary figures and images for: Slow light topological photonics with counter-propagating waves and its active control on a chip
Source: Nat Commun. 2024 Jan 31;15:926. doi: 10.1038/s41467-024-45175-5 (PMC10830473; doi:10.1038/s41467-024-45175-5)

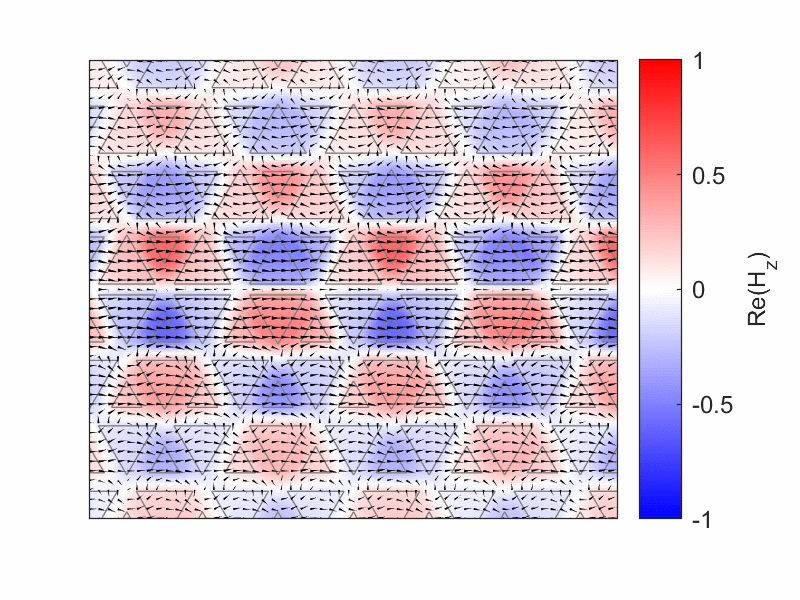

Supplement: Supplementary file 4 — Supplementary Movie 1 [file 41467_2024_45175_MOESM4_ESM.gif]

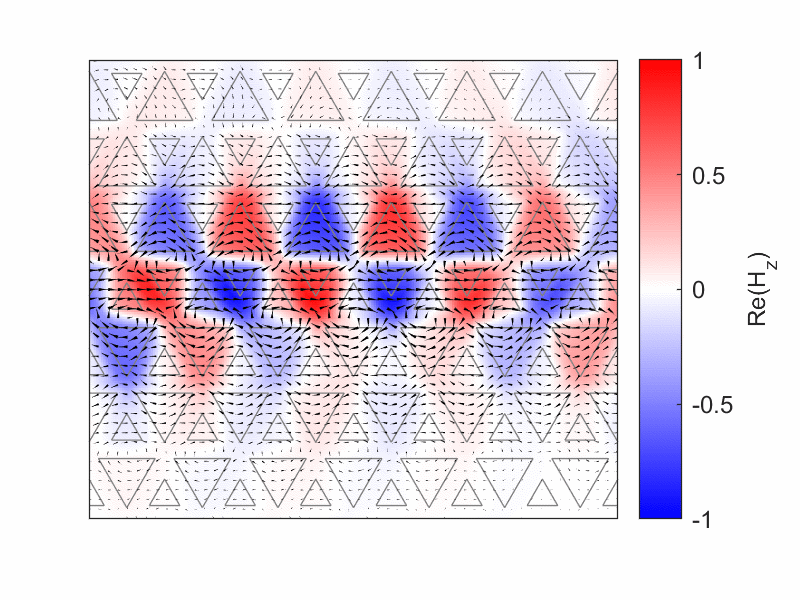

Supplement: Supplementary file 5 — Supplementary Movie 2 [file 41467_2024_45175_MOESM5_ESM.gif]

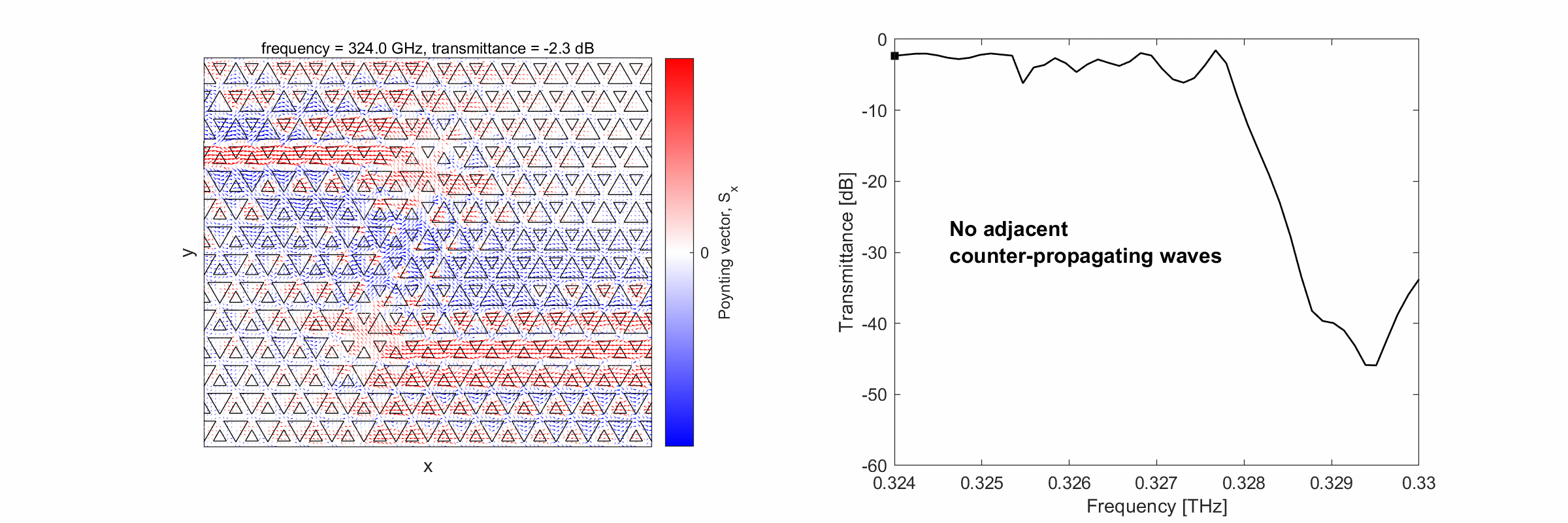

Supplement: Supplementary file 6 — Supplementary Movie 3 [file 41467_2024_45175_MOESM6_ESM.gif]
